# Innovative microbial strategies in atopic dermatitis

**DOI:** 10.3389/fimmu.2025.1605434

**Published:** 2025-07-23

**Authors:** Jingtai Ma, Yiting Fang, Jinxing Hu, Shiqi Li, Lilian Zeng, Siyi Chen, Zhifeng Li, Ruiling Meng, Xingfen Yang, Fenglin Zhang, Guiyuan Ji, Peihua Liao, Liang Chen, Wei Wu

**Affiliations:** ^1^ Guangzhou Chest Hospital, Guangzhou, China; ^2^ Guangdong Provincial Institute of Public Health, Guangdong Provincial Center for Disease Control and Prevention, Guangzhou, China; ^3^ Guangdong Provincial Center for Disease Control and Prevention, Guangzhou, China; ^4^ National Medical Products Administration (NMPA) Key Laboratory for Safety Evaluation of Cosmetics, Guangdong Provincial Key Laboratory of Tropical Disease Research, School of Public Health, Southern Medical University, Guangzhou, China; ^5^ Guangzhou Institute of Microbiology Group Co., Ltd., Guangzhou, China; ^6^ Xinjiang Uighur Autonomous Region Center for Disease Control and Prevention, Uighur, China; ^7^ Zhuhai Center for Disease Control and Prevention, Zhuhai, China

**Keywords:** atopic dermatitis, microbial strategies, fecal microbiota transplantation, probiotics, postbiotics, prebiotics, CpG-ODNs, herbal fermentation technology

## Abstract

Atopic dermatitis (AD) is characterized by chronic and recurrent itching with a high burden of disability-adjusted life years (DALYs, a measure of overall disease burden). Traditional treatments mainly include corticosteroids, which have a good effect on controlling inflammation but adverse side effects. Recently, advancements in understanding the pathogenesis of AD have led to the emergence of a variety of novel therapeutic approaches, such as microbiome manipulation, offering renewed hope for more effective management of this condition. These strategies are particularly promising for mild-to-moderate AD, where dysbiosis and immune imbalance (e.g., Th2 skewing) are key drivers, though some approaches (e.g., fecal microbiota transplantation) are being explored for refractory cases. It has been shown that microbiome manipulation has the potential to improve disease states and regulates the balance of the inflammatory system in a variety of ways. Various approaches have been preclinically and clinically tested, including probiotics (and multiple co-applications), prebiotics, postbiotics, unmethylated CpG motifs, fecal microbiota transplantation, herbal fermentation technology with microorganisms and phage. In this review, we discuss these microbiome manipulation methods and emphasizes the potential of microbiome-based interventions to modulate Th1/Th2 balance with fewer side effects, ultimately leading to control of inflammation in AD. Further translational research in this field is needed to integrate when we apply this therapy and the capability for disease treatment and prevention.

## 1 Atopic dermatitis

Atopic dermatitis (AD) is a chronic, recurrent, inflammatory and pruritic dermatosis with complex pathophysiology, involving disruption of the epidermal barrier, microbial dysbiosis within affected lesions, and Th1/Th2-imbalanced immune responses to skin allergens (1). AD is a highly heterogeneous chronic inflammatory skin disease, and its clinical manifestations and etiology vary significantly among different age groups. In infancy, AD is mainly acute eczema; in childhood, AD is mainly flexor dermatitis; in adolescence and adulthood, AD presents as chronic lichenification; while in old age, AD shows a “reverse” distribution feature. AD in different age groups varies in terms of genetics, immunity, environment and psychological factors, but pruritus, skin barrier dysfunction and immune system abnormalities are the common pathological basis (2–4). AD has the highest DALY burden of all skin diseases and ranks 15th among all nonfatal diseases globally. The global burden of disease Study estimated that the general prevalence of AD was 15-20% among children and up to 10% among adults (5). Although most AD cases are mild-to-moderate (6), this condition is characterized by severe pruritus, which frequently results in skin injury, considerable sleep disruption, and a detrimental impact on the overall quality of life (7).

**Figure 1:**
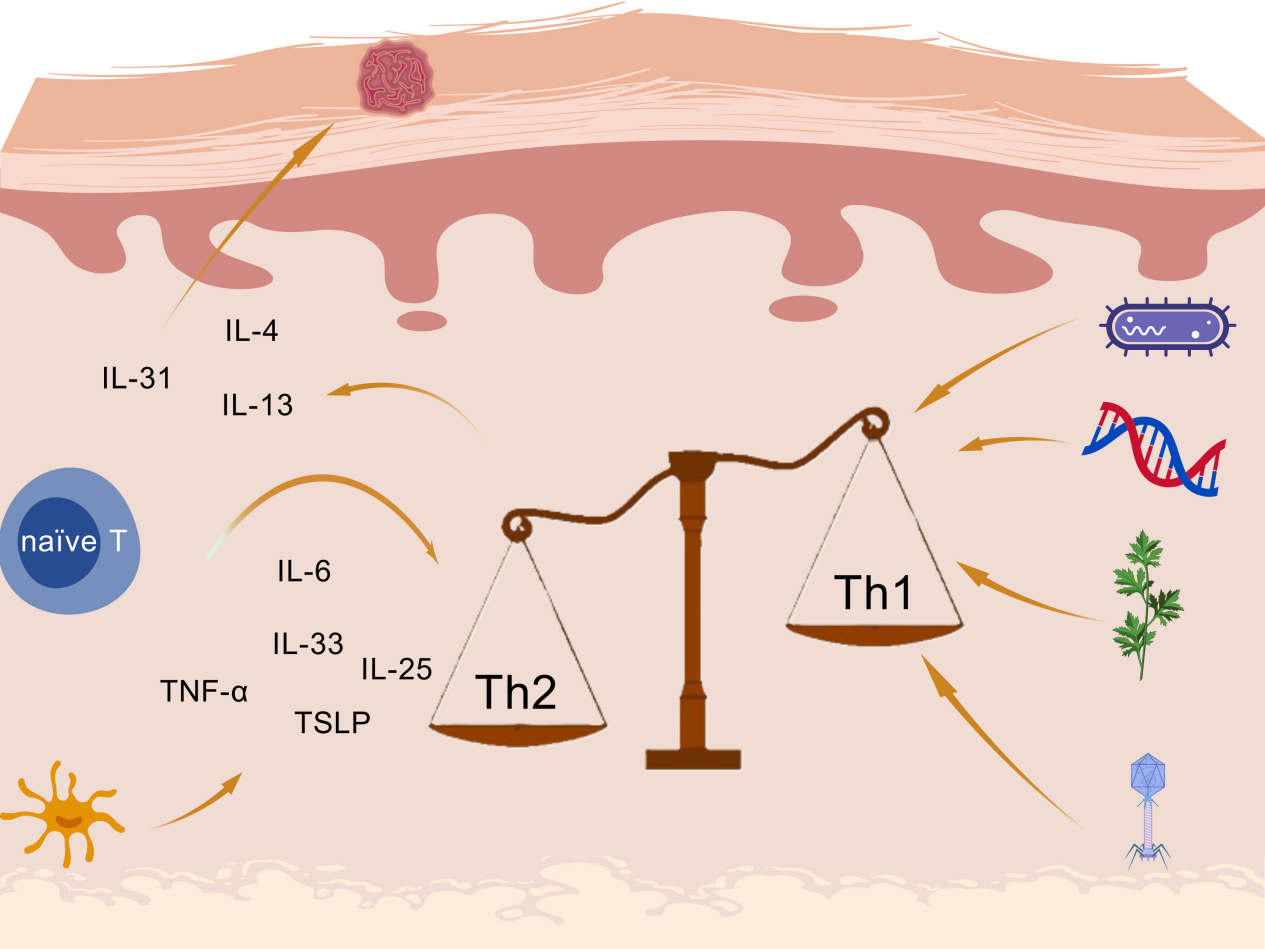
Inflammatory mechanisms in atopic dermatitis created with BioGDP.com (98).

The exact mechanism underlying the complex progression of AD is not yet well understood. However, after a continuous exploration, current evidence indicates that the mechanism is related to genetic abnormalities, immunological dysfunction (mainly Th1/Th2 imbalance caused by excessive activation of Th2 immunity), and environmental factors as the prime drivers of AD exacerbation (8), as shown in **Figure 1**. Those factors contribute to skin barrier dysfunction and allow entry of external antigens and allergens, which activate immune responses across the skin surface.

Under allergen conditions, pro-inflammatory cytokines including interleukin (IL)-6, IL-25, IL-33, TNF-α, and thymic stromal lymphopoietin (TSLP) are produced by keratinocytes and Langerhans cells (LCs), leading to the activation of the Th2 immune axis through type 2 innate lymphoid cells (ILC2) and Th2 cells differentiate from naïve CD4+ T cells (9). Janus kinase (JAK) pathway is also activated, leading to a variety of cytokines and the enhance of Th2 cell differentiation (10, 11). The imbalanced and hyperactivated Th2-type immune responses induce the isotype class switch from IgM to IgE by mast cells, triggering a hypersensitivity reaction. Th2 cells secrete cytokines: IL-4 and IL-13 contribute to skin barrier defects, cutaneous infections, inflammation, thickening, and itching (12); IL-31 accounts for the stimulation of itch receptors, ultimately leading to pruritus in AD (13, 14).

Furthermore, increased Th2 polarization in AD not only disrupts the balance between Th1 and Th2 cells through excessive Th2 activation, but also contributes to pruritus. In response to itching, frequent scratching damages the skin barrier, increasing sensitivity to minor stimuli and facilitating the entry of external antigens and allergens such as dust. This, in turn, leads to further inflammation and itching, creating a vicious cycle.

## 2 Conventional and emerging therapeutics for atopic dermatitis

The management approaches for AD ought to be customized according to the unique attributes and advancement of the illness in each patient, with a primary focus on inflammation control. In most instances, corticosteroids and calcineurin inhibitors can be localized to the affected area and are widely used in the treatment of AD.

Topical corticosteroids (TCS) are widely recognized as first‐line anti‐inflammatory agents and are grouped into 7 classes, based on potency (15). This relies heavily on the physician’s experience and peer communication to adjust medications to balance efficacy and side effects (e.g. skin atrophy, purpura, telangiectasia, hypopigmentation, focal hypertrichosis, acneiform eruptions, and striae) (8). Topical calcineurin inhibitors (TCIs) exert their anti-inflammatory effects by inhibiting the activation and proliferation of calmodulin-dependent T cells (16), which do not cause skin atrophy but itching (17).

As for patients with chronic and severe AD, topical therapies are often insufficient for disease control. In such cases, systemic treatments, including oral and subcutaneous administration of medications (such as immunosuppressants and biologics), are required to manage symptoms and reduce disease severity (18, 19). Systemic corticosteroids are reserved for short-term use in acute flares due to risks like adrenal suppression and metabolic complications (20). Non-specific immunosuppressants like cyclosporine and methotrexate reduce inflammation by suppressing the immune response, widely employed in patients with refractory AD (21). Due to systemic administration, prolonged use of the above drugs can result significant adverse effects: cyclosporine is associated with nephrotoxicity (17); methotrexate is linked to hepatotoxicity (22). Therefore, careful monitoring is necessary during long-term treatment, and there is an urgent need for safer, long-term therapeutic options with fewer side effects.

Biologic therapies, particularly dupilumab, a monoclonal antibody targeting IL-4 and IL-13 signaling, have transformed treatment for moderate-to-severe AD (23, 24). Dupilumab is generally well tolerated and has shown sustained improvements in symptoms and quality of life (25). Additionally, JAK inhibitors (e.g., baricitinib, upadacitinib, abrocitinib) offer oral alternatives by interfering with cytokine signaling involved in inflammation (26–28). These agents are effective in moderate-to-severe AD but may carry risks such as infections, lipid elevations, and laboratory abnormalities, warranting individualized risk-benefit assessment (27, 29, 30).

Therefore, challenges remain — including drug-related adverse effects, long-term safety concerns, and high costs — underscoring the need for safer, more sustainable alternatives. In this context, microbiome-based therapies have gained attention for their potential to modulate immune responses and restore microbial balance with generally favorable tolerability.

**Figure 2:**
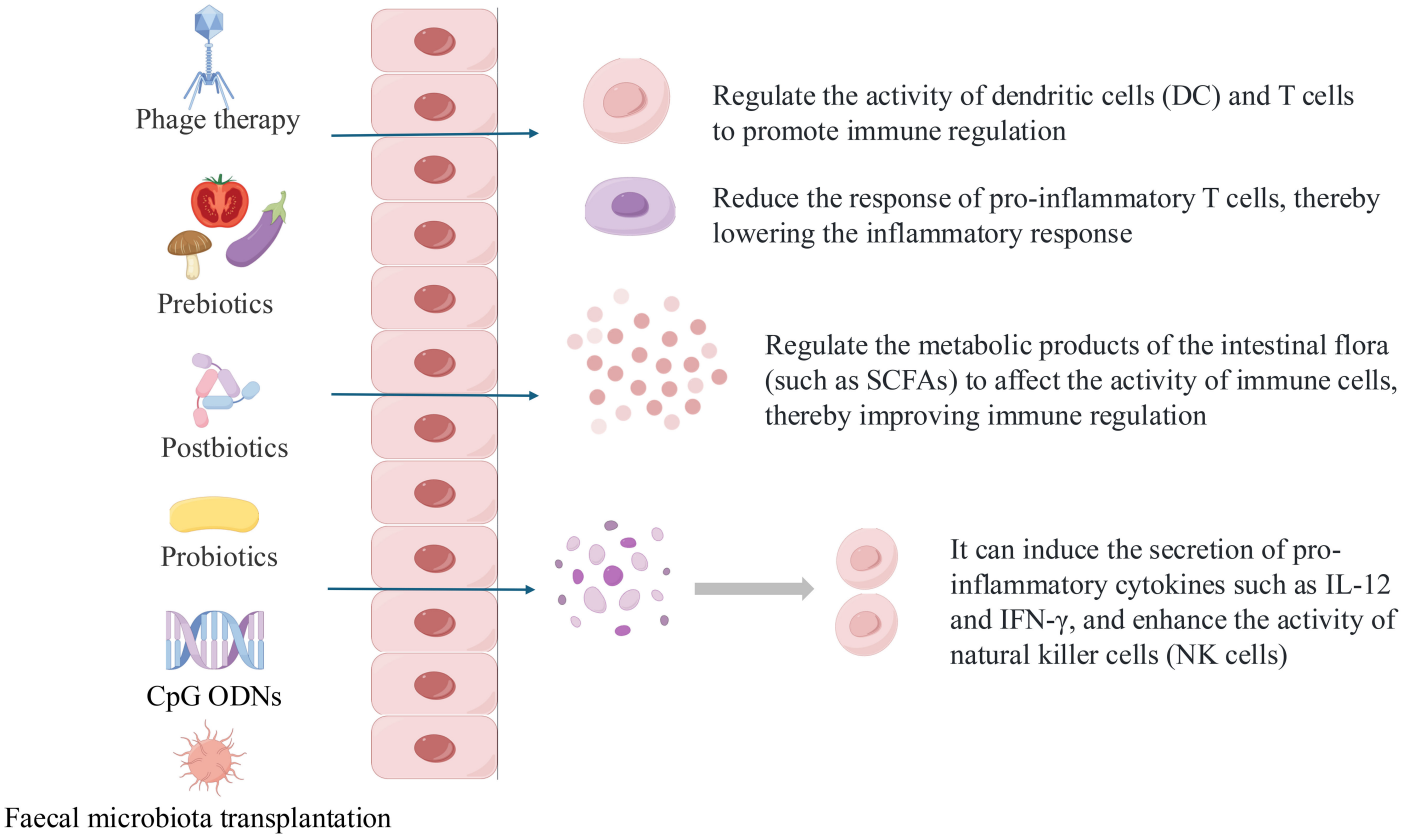
What are the possible mechanisms of microbiome operation methods and how do they affect humoral and cellular immunity.

Such approaches — including probiotics, prebiotics, postbiotics, phage therapy, and microbiota-derived compounds — are particularly promising for mild-to-moderate AD and may complement existing treatments or serve as preventive strategies, as shown in **Figure 2**. By addressing underlying dysbiosis, these interventions represent an innovative and evolving therapeutic avenue in AD management.

## 3 Microbiome and AD

The human microbiome is composed of a collection of dynamic microbial communities (31). Accordingly, the balance of gut microbial environment has resulted in these communities playing a profound role in promoting human health and disruptions (mainly decreased bacterial diversity) may contribute to inflammation and susceptibility to allergy. Pretreatment with antibiotic combinations can eliminate pathogens, especially predominant pathogenic bacteria in dysbiosis and enhance the anti-inflammatory effects of probiotics by reducing pro-inflammatory cytokines and improving histological injury (32). Antibiotic-induced dysbiosis of intestinal flora enhances influenza virus damage to lung and intestinal mucosa and affects Th1/Th2 balance (33). Intervention studies have shown that probiotics improve intestinal dysbiosis in bronchial asthma (allergy), improve elevated serum IgE, IL-4, IL-5, IL-9, and IL-13 levels, and decrease Th1/Th2 ratio in asthmatic children (34). In the gut microbiota of AD patients, there is a lack of *Bifidobacterium* (except *B. pseudocatenulatum*), *Enterococcus*, *Bacteroides*, and *Ruminococcus* genus, while an overabundance of *Staphylococcus aureus* (*S. aureus.*), *Clostridium* spp. and *Escherichia coli (* 35, 36).

The skin microbiome is a highly characterized ecosystem that varies significantly across different skin surfaces. Correspondingly, in the skin microbiota, there is an overabundance of *S. aureus.*, *S. epidermidis*, and *S. haemolyticus (* 37). In addition to overgrowth, *S. aureus.* strains in AD patients often exhibit high levels of antibiotic resistance. Studies have shown that patients with AD have significantly higher resistance rates to antibiotics such as penicillin, erythromycin, chloramphenicol, and tetracycline compared to the general population. For example, in a study involving 194 *S. aureus* strains, resistance to oxacillin was 1.4% in the AD group versus 0% in controls; erythromycin resistance was 45.1% in AD patients versus 23.4% in controls. These findings underscore the challenges in treating secondary infections in AD and highlight the importance of understanding microbial ecology and resistance patterns (38). Animal studies have demonstrated that changes in skin microbiota modulated immune cell dynamics, restoring the Th1/Th2 balance and leading to clinical improvement (39).

Recently, advancements in understanding the pathogenesis of AD have led to the emergence of a variety of novel therapeutic approaches, offering renewed hope for more effective management of this condition. Traditional therapies are associated with high side effects, as well as a lack of treatment and prevention for mild-to-moderate AD. In this review, we explore microbiome-based therapeutic strategies for AD and provide an updated perspective on chronic skin inflammation management. We focus on identifying treatment approaches that combine strong clinical efficacy with favorable safety profiles and high patient acceptability—attributes particularly important in chronic conditions like AD where long-term adherence is essential. The potential high acceptability of microbiome-targeted therapies stems from their non-invasive nature, avoidance of systemic immunosuppression, and alignment with growing patient preference for “natural” or physiology-modulating treatments over conventional pharmacotherapies.

## 4 Fecal microbiota transplantation

Fecal microbiota transplantation (FMT), a novel strategy for gut microbiota-induced disorders, allows long-lasting alteration of the recipient’s microbiome and temporary colonization to achieve the purpose of treatment. Briefly, intestinal probiotics are transplanted from healthy volunteers to receptors with gut microbiota disturbance, thus yielding the microecological balance in the rebuilt gut microbiota. The potential therapeutic effect of FMT was investigated by gut microbial ecology, immune system modulation, and fecal metabolite analysis using AD mice models.

After establishing AD mouse models, fecal microbiota transplantation (FMT) was found to reduce dermatitis scores compared to the negative control group (40, 41). Alpha diversity in the FMT group was initially lower than that of the donor group but increased following FMT treatment. Notably, the recovery of the gut microbial community showed significant variation starting from week 7 (40). In β-diversity analysis, there was no significant difference between the FMT and donor groups (41), whereas the negative control group showed a significant difference after just one week (42).

In further detail, at the phylum level, Firmicutes increased, while Bacteroidetes decreased in the AD model mice (41, 42), resulting increased the levels of SCFA (acetic, butyric, isobutyric, and propionic acid) levels (40). At the genus level, the abundance of Lactobacillus was increased in the FMT-treated groups compared with the negative control group (40, 42).

In the regulation of inflammatory factors, serum levels of IgE (40, 41) and IgG1 (41) were significantly decreased compared with the negative control group. The concentrations of Th2 cytokines (IL-4, IL-5, and IL-13), which are known to contribute to the development of AD, were significantly decreased compared with the negative control group after 7 weeks (40) and 2 weeks (41), and a study has reported that IL-4 and IL-13 were significantly decreased in 1 weeks (42). However, the concentrations of Th1 cytokines, such as IL-12, IFN-γ, and TNF-α, were significantly increased in the FMT group than in the negative control group (40).

FMT modulated Tregs through enhanced their surface PD-1 levels and Helioxs PD-1 Tregs levels and reduced cytokines levels secreted by Tregs (i.e., IL-10 and IL-1β) (41). Further mechanistic studies revealed that FMT treatment induced CD103 DCs and programmed cell death ligand 1 (PD-L1)/programmed cell death 1 (PD-1) expression in skin-draining lymph nodes and promoted Treg production to induce immune tolerance and suppress skin inflammation.

## 5 Oral supplements

Direct oral supplementation is another and more acceptable way to modulate the gut microbiota and microbial environment including probiotics, postbiotics, prebiotics and other products. The results of the randomized controlled trial showed a good effect, significantly reducing dermatitis scores and relieving itching. However, the pathway how achieves its effects of modulating inflammation remain a focus of research. The following explores the pathways of inflammation modulation and the development of new formulations.

### 5.1 Lactobacillus

In recent years, there has been more development and research on *Lactobacillus* and it has anti-allergic effects and modulating Th1/Th2 balance in AD models. A new train of the bacterium, *Lactobacillus acidophilus* (*L. acidophilus*) KBL409 reducing in serum IgE and regulating the mRNA levels of Th1 (Interferon-γ) and Th2 (IL-4, IL-5, IL-13, and IL-31) cytokines in skin tissues (43). Otherwise, Th17 (IL-17A) cytokines, anti-inflammatory cytokine IL-10 and the expression of Forkhead box protein P3 (Foxp3) were also increased.

A new strain, *Lactocaseibacillus paracasei subsp. paracasei NTU 101* (NTU101), with similar effects including effectively alleviating skin symptoms and reducing the infiltration of inflammatory cells and the potential mechanism involves increasing the expression of Foxp3, with the same pathway about Foxp3 in the above (44). Further study verified NTU 101,and bacteria of the same genus *Lactobacillus paracasei KBL382*, influenced the proportion of CD4+CD25+Foxp3+ (Treg) cell to promote immune tolerance, and modulated the immune response by increasing the proportion of CD4+/IL-4+ (Th2) cells in the spleen and CD4+/IFN-γ+ (Th1) cells, achieving a rebalancing of the Th1/Th2 ratio (45, 46).

In the process of fractionating culture supernatants of *Lactobacillus gasseri (L. gasseri)*, Chen selected a moonlighting protein, glycolytic glyceraldehyde 3-phosphate dehydrogenase (LGp40) and it can enhance skin barrier function and reduced infiltration of Langerhans cells in the dermis, and mitigated skewed Th2 and Th17 immune responses (47). Potential mechanisms include inhibiting allergen-induced keratinocyte apoptosis through the blockade of the caspa-se-3 cascade and reducing the NLR family pyrin domain containing 3 (NLRP3) in-flammasome in macrophages to regulate inflammation (48).

### 5.2 Lactiplantibacillus plantarum

*Lactiplantibacillus plantarum* (*L. plantarum*) is widely used in the pharmaceutical industries. Recent study found that the strains *L. plantarum* HD02 and MD159 demonstrated comparable preventive effects against allergies, reducing blood levels of mast cell protease-1 (MCPT-1) and total IgE (49). L. plantarum APsulloc 331261 downregulated Th2 expression, and upregulated Th1 expression in a colony-forming unit-dependent manner, showing its effect of relieving AD symptoms (50).

### 5.3 Pediococcus

By transforming dendritic cells into tolerogenic versions, *Pediococcus (pentosaceus KF159)* directly enhanced the production or secretion of IL10 through the induction of CD4+CD25+Foxp3(Treg) cells, which performed similarly to NTU101 (51) and *Pediococcus acidilactici SRCM102024* dose-dependently improved the clinical symptoms similar to the positive-control (dexamethasone 3 mg/kg bw) and had fewer adverse effects (52).

### 5.4 Bifidobacteria

Past reviews have summarized the therapeutic effects of *Bifidobacteria* on atopic dermatitis: *B. longum* can reshape gut microbial composition and upregulated tryptophan metabolism of gut microbiota to increase I3C, which medicated AHR signaling pathway to improve clinical symptoms in AD combined metagenomic sequencing analysis and targeted tryptophan metabolic analysis (53). While considerable research has focused on exploring single bacterial species or consortia, the optimal strategies for microbiota-based therapeutics remain underexplored. Specifically, the comparative effectiveness of bacterial consortia versus individual species warrants further investigation. One of the innovative Strategies for Bifidobacterium is to combine with other Probiotics.

Two studies assessed the impact of bacterial consortium included *L. plantarum*, *Bifidobacterium* and *Lacticaseibacillus casei*, due to their synergistic effect on IL-10 production, an anti-inflammatory cytokine that plays a pivotal role in modeling the gut and skin microbiome: One found that the administration of bacterial consortium demonstrated enhanced therapeutic efficacy in experimental models(mice) of atopic dermatitis when compared to single strains. Bacterial consortium significantly restrained detrimental inflammatory immune responses, including reduced ear swelling, suppressed immune cell infiltration, and decreased serum immunoglobulin E(IgE) levels. These effects are likely attributed to the upregulation of intestinal regulatory CD103+CD11b+ dendritic cells and alterations in the microbiome communities (54); Another Randomized Controlled Trial (RCT) evaluated the therapeutic efficacy of a bacterial consortium in AD patients. The results demonstrated significant reductions in all clinical severity scores, including erythema, edema/papulation, excoriation, total inflammation score (TIS), and PRURISCORE, both at the group level and within individual patients. However, current evidence remains insufficient to determine whether these improvements result from synergistic effects among the bacterial strains, as no studies have specifically investigated this mechanistic aspect (55).

A new formulation of probiotics, which composed of one strain of *Bifidobacterium* and four strains of *Lactobacillus*, containing 5x10^10^ CFU active probiotics per gram, has been explored the efficacy in the treatment of AD and found that the formulation significantly alleviated skin inflammation of the MC903-induced AD in mice. Administration with the probiotics mixture induced increased production of regulatory T cells and regulatory dendritic cells (DCregs) in the mesenteric lymph nodes, thus modulating the immune response (56).

A new formulation of probiotics, YK4, including comprising *Lactobacillus acidophilus CBT LA1*, *L. plantarum CBT LP3*, *Bifidobacterium breve CBT BR3*, and *B. lactis CBT BL3* and it regulates Th1/Th2 balance and suppresses inflammation through DCs, Tregs and Galectin-9 in DNCB-induced mice, indicating a potential role as an effective anti-inflammatory agent in AD patients (57).

### 5.5 Postbiotics

Defined by the International Scientific Association of Probiotics and Prebiotics (ISAPP) as preparations of inanimate microorganisms and/or their components that confer health benefits. Postbiotics include heat-killed bacteria, cell-free supernatants, and purified microbial components that maintain the beneficial properties of probiotics while ensuring safety and stability (58). As a high-temperature inactivated microbial preparation, postbiotics have the property of being easy to store and more acceptable compared to probiotics, but may only be able to exert part of the effect in relieving and treating inflammation.

A new postbiotic product has been reported and it can rebalance the population of Th1/Th2 cells in the spleen of HDM extraction-induced AD mice and was possibly related to modulate the proportion of IL-4+ CD4+ T cells and IFN-γ+ CD4+ T cells (59). Moreover, it has capability of decreasing serum IgE concentration to relieve the classic AD signs and the production process includes isolating two lactic acid bacteria strains (*Lactococcus lactis subsp. cremoris MP01* and *Lactobacillus paracasei subsp. paracasei MP02*) from traditional fermented milk and being heat inactivated.

The recent review suggested that *Lactobacillus* postbiotics might be successfully used as adjuvant AD therapy in adults. Thus far, data do not indicate efficacy in pediatric patients. This age-dependent difference can be explained by three key factors observed in probiotic studies: different immunomodulatory mechanisms between the mature and developing immune systems, strain/dose response changes, and different stages of gut microbiota development (60–62). Standardizing nomenclatures and experimental procedures, as well as expanding the studies to more geographic locations and assessing comprehensively the effects on the gut microbiome would provide better perspectives of postbiotics as a therapeutic option for AD (63).

### 5.6 Prebiotics

Prebiotics are fermented ingredients that alter the composition and activity of the gut microflora, providing health benefits to the host. Xylooligosaccharide (XOS) is similar to fructo-oligosaccharides (FOS) and anti-inflammatory effects are achieved by promoting the reproduction of bifidobacteria. Laigaard et al. (64) addressed the effects of a prebiotic, XOS, on the gut microbiota and ear inflammation in an oxazolone-induced AD model. Feeding XOS alleviates oxazolone-induced hyperresponsiveness in AD mice. Serum IgE and ear tissue cytokine levels correlated significantly with the clinical scores. The prebiotic treatments (e.g. scGOS/lcFOS, inulin, and β- glucan) showed suppression of AD symptoms (65), Th2 cell differentiation, and AD-like skin lesions induced by DNCB and were immunomodulated via activation of galectin-9 and toll-like receptor 9 (TLR-9) (66).

## 6 CpG-ODNs

CpG oligodeoxynucleotides (CpG ODNs) are the artificial versions of unmethylated CpG motifs which were originally discovered in bacterial DNA, and demonstrated not only as potent immunoadjuvants but also as anticancer agents by triggering TLR9 activation (67). In humans, TLR9 is highly expressed in plasmacytoid DCs and B cells (68). CpG-ODN in high amount activates a TLR9-TRIF-non canonical NFκB-IRF3 cascade to mediate anti-inflammatory molecules such as IL-10, TGF-β and IDO (69). Consequently, such treatment at high dose combined with allergens could be an innovative strategy for the development of Tregs and B regulatory (Breg) cells during allergen-specific immunotherapy (AIT) protocol (70) such as high-dose CpG in combination with allergens without endotoxin toxicity to boost immune tolerance (71).

CpG-ODNs are strong Th1 response inducers while inhibiting Th2 responses, helping restore the balance of Th1/Th2. Studies have reported that CpG-ODNs prevent T helper 2 (Th2) allergic responses such as antigen-induced asthmatic responses, allergic rhinitis and AD (72) and trigger Th1 response via induction of IFN-α and-γ, as well as IL-12 to restore the balance of Th1/Th2. In addition, they can inhibit IgE production in B cells (73). Therefore CpG-ODNs are considered as adjuvant treatment for T helper 2 (Th2) allergic responses such as antigen-induced asthmatic responses, allergic rhinitis and AD (72).

Given that *Bifidobacterium* have specific immunostimulatory properties that influence the Th1/Th2 balance, these probiotics may have therapeutic potential for AD. A similar methylated CpG site capable to activate the *in-vitro* Treg cell differentiation was identified in the genomic DNA from the probiotic *B. longum subsp. Infantis* (74) and compared to *Lactobacillus*, *Bifidobacterium* have higher more CpG motifs (75).

Kim et al. (76) focused on the effects of three types of *B. bifidum* (probiotics, postbiotics, CpG ODN) and found that oral administration of the three types of *B. bifidum* alleviated the clinical symptoms of AD in the skin lesions by modulating the balance of Th1/Th2, Treg/Th1 and Treg/Th1+Th2.

Synthetic CpG-ODNs, which contain a partial or complete phosphorothioate (PS) backbone, exhibit immunotoxicity and hepatotoxicity, leading to conditions such as splenomegaly, liver necrosis, and hemorrhagic ascites (77). A low-toxic CpG-ODN with a phosphodiester backbone was synthesized and designated as 46O, and it suppressed IgE and IL-4 synthesis in mice with OVA-induced AD (78).

## 7 Herbal fermentation technology with microorganisms

Herbal fermentation technology with microorganisms refers to the process of culturing herbs with microorganisms to enhance their function and promote their absorption (79) and is expected to be the link between modern medicine and traditional Chinese medicine.

Compared with unfermented Portulaca oleracea L. (PO), PO fermented using 1% (v/v) *Bacillus* sp. *DU-106* and 1% (v/v) *Lactobacillus plantarum*. improved the skin barrier in AD mice and limited the expression of inflammatory cytokines and exerts an anti-inflammatory effect by suppressing NF-κB signaling pathway and outperformed in reducing inflammation (80). Another study accessed postbiotics fermented from Smilaxchina L. leaves and *Lactobacillus acidophilus* (KCTC15475BP), indicating that the postbiotics significantly alleviated AD symptoms and suppressed the AD response by effectively regulating chemokines and cytokines through the reduction of NF-κB activity driven by inflammation (81). After probiotic fermentation, Morinda citrifolia significantly alleviated AD symptoms such as infiltration of inflammatory cells (e.g., mast cells and eosinophils), reduced the levels of IgE, TSLP and beneficially modulated the expressions of Th1, Th2(, Th17, and Th22)-mediated cytokines in lesioned skin and splenocytes (82).

## 8 Bacteriophages

Bacteriophages, also known as phages, are a type of virus that selectively target and infect bacteria (83). Compared with antibiotics, phages have stronger host specificity, which do not destroy the normal microflora of human body (84) and do have a profound impact on the bacteria that exist within and on humans.

Byrd et al. (85) analyzed microbial temporal dynamics from a cohort of pediatric AD patients sampled throughout the course of disease by Shotgun metagenomic sequence analysis and found that heterogeneous *S. aureus.* and *Staphylococcus* epidermidis are closely related to AD. Wang et al. (86) employed a random forest classifier to identify potential marker genes and highlighted the genetic diversity and functional implications of prophages in driving differentiation between strains from AD and healthy control group. Moreover, prophages in the healthy control group exhibited variously higher enrichment of differential functions and the AD group displayed a notable enrichment of virulence factors within their prophages, underscoring the important contribution of prophages to the pathogenesis of AD-associated strains.

Phage therapy has a theoretical basis to be an innovative solution for treating skin inflammation caused by certain specific strains of bacteria (87) and in recent years, lytic phages targeting specific bacterial groups (*S. aureus.*) are being developed and applied in humans.

Phage SAP71, which specifically lysed S. aureus, was able to significantly reduce inflammatory cell infiltration, and prevent the development of AD-like skin pathological changes in an AD model (88). Meanwhile, the bacteriophage-surfactant combination could increase the eradication of IgE-stimulated aggregation *in vitro* test, and significantly decolonized the pathogen with an efficacy double that of the phage-only treatment, and decreased the expression of pro-inflammatory cytokine genes (IL-1β, IL-12 and IFN-γ) for 5 days *in vivo* trial (89).

In summary, phage therapy has the potential to act as an alternative to antibiotics for *S. aureus* decolonization in patients with dermatitis and contribute to combat the antibiotic resistance.

## 9 Conclusion

This review has outlined the current landscape of microbiome-based therapies for AD, emphasizing their potential to modulate immune responses, restore microbial balance, and reduce inflammation. Approaches such as FMT, probiotics, prebiotics, postbiotics, phage therapy, and probiotic-fermented botanicals demonstrate promising results in reshaping the host microbiome and managing AD symptoms.

FMT has shown efficacy in modifying gut microbiota and is being explored in complex diseases such as autism and multiple sclerosis (90). However, its application in AD remains experimental and requires standardization in terms of microbial composition, dosage, formulation, and administration methods.

As research progresses, probiotics, prebiotics, postbiotics, and related products are increasingly applied in allergic diseases, including eczema. These interventions are generally considered well-tolerated based on existing studies, although comprehensive evaluations of adverse effects are still limited and further investigation is warranted.

Different reagents have different applications: Probiotics, is a fermented product that is expected to be used to modulate intestinal microbiota and maintain immune homeostasis in milk powder or other reagents. As the source of probiotic products, development of new strains remains a priority. It is also required to further explore the pairing of different flora and a more detailed mechanism. Although with a longer shelf life and lower biological activity than probiotics, postbiotics can be used for safe pharmaceutical and food-manufacturing applications. CpG ODNs, as potent immunoadjuvants, are being developed for different diseases, such as nanomedicines for tumor and nebulization therapy for asthma (91, 92). In the future, a potential therapeutic approach for AD could involve an ointment containing CpG ODNs, although this remains speculative and requires experimental validation.

Recent study has proved that probiotic-fermented herbs are able to alleviate AD symptoms and regulate Th1/Th2 balance in mice. PO has antioxidant, allergic and inflammatory effects. After fermentation, beneficial effects were observed along with elevated polyphenol and flavonoid content, indicating that it may work by increasing the beneficial phytocompounds. Through modern processing technology, we can not only enhance the beneficial effects of the original drugs (especially traditional Chinese medicine), but also help us to explore the mechanism.

Phage therapy is similar to antibiotic therapy in that it kills harmful flora (specifically *S. aureus.*) and reduces the inflammatory response, which also means that there is the problem in restoration of skin flora. In addition, the proteins and nucleic acids of the phage, as well as the excipients used in the administration of the drug, need to be taken into account to determine whether they are allergenic. Moreover, attention needs to be paid to whether the phage proteins, nucleic acids and pharmaceutical excipients can trigger allergies.

Based on the current research, the optimal treatment for mild-to-moderate AD includes TCS to rapidly control inflammation, combined with microbiome-based therapies to restore a healthy skin microecology, which may prove to be a useful therapeutic paradigm. In addition to TCS, calcineurin inhibitors play a crucial role in managing AD, especially in sensitive skin areas, and their use may complement microbial interventions aimed at restoring immune and microbial balance (93). The further goal is to use microbial therapies early and appropriately to reduce the use of hormonal medications and even to prevent the onset of AD. Beyond their application in atopic dermatitis, many of the microbial therapies discussed—such as probiotics, CpG-ODNs, and fecal microbiota transplantation—are being investigated for a broad spectrum of chronic inflammatory and immune-mediated diseases. For instance, FMT has shown promising results in conditions like ulcerative colitis, multiple sclerosis, and even autism spectrum disorders, highlighting the central role of the gut–immune axis (94–96). Similarly, CpG-ODNs are under development as immunotherapies for asthma and certain cancers due to their potent ability to shift Th2-skewed immunity toward a Th1-dominant response (40, 97). The success of these strategies in AD could pave the way for cross-condition translational therapies, especially in diseases that share common immunopathological features such as Th2 polarization or microbial dysbiosis. Future studies comparing microbial interventions across diseases may reveal shared microbial signatures and therapeutic targets, accelerating the design of more personalized and preventive treatments. Integrating microbiome-based strategies into a broader immunological framework could ultimately redefine how we treat not only skin disorders but systemic inflammatory diseases as well.
